# Development of skin hypopigmentation in a patient with metastatic papillary carcinoma thyroid treated with Sorafenib

**DOI:** 10.1186/1472-6823-13-29

**Published:** 2013-08-09

**Authors:** Syed Zubair Hussain, Ali Asghar, Mubasher Ikram, Najmul Islam

**Affiliations:** 1 Department of Medicine, Section of Endocrinology, Aga Khan University Hospital, Stadium Road, P.O. Box 3500, Karachi 74800 Pakistan; 2 Department of Surgery, Section of Otorhinolaryngology, Aga Khan University Hospital, Stadium Road, P.O. Box 3500, Karachi 74800 Pakistan

**Keywords:** Thyroid Carcinoma, Sorafenib, Hypopigmentation

## Abstract

**Background:**

Sorafenib can be considered as the effective option of treatment in patients with metastatic radioiodine refractory differentiated thyroid cancers. The cutaneous manifestations of Sorafenib include rash, desquamation, hand foot skin reactions, pruritus, alopecia and erythema. We report the first case of hypopigmentation related to sorafenib therapy.

**Case presentation:**

We report the case of a middle aged gentleman with metastatic papillary carcinoma of thyroid diagnosed in 2005. He was managed with total thyroidectomy, radioactive iodine and TSH suppressive therapy. Despite receiving radioactive iodine 530 mci cumulative dose, patient had persistant disease with lung metastasis. Therefore a TKI, sorafenib, was started. He developed hypopigmentation of the skin more prominent on face six weeks after starting sorafenib treatment.He also developed diarrhea, desquamation of hands and feet, hair loss over scalp, eye brows and moustache. Sorafenib treatment was discontinued. His diarrhea stopped in one week and after four weeks his skin became normalized whereas he regained his hairs in six weeks.

**Conclusion:**

To our knowledge, hypopigmentation in our patient appears to be the first reported of its kind in the literature to date. Sorafenib is used in Renal cell carcinoma, Hepatcellular carcinoma and radioactive iodine refractory thyroid carcinoma therefore it is very important to be aware of hypopigmentation as a potential side effect for both physicians and patients.

## Background

Thyroid cancer is the most common and prevalent endocrine malignancy [1,2]. It accounts for about 95% of endocrine malignancies [2]. The most common type of thyroid cancer is differentiated thyroid cancer (DTC) 95%, which arise from the follicular cells of the thyroid and includes papillary and follicular subtypes. Treatment of differentiated thyroid cancer includes surgery, radioactive iodine(I^131^) and TSH suppressive therapy. The prognosis in DTC is usually good as these tumors are slowly progressive and frequently curable with conventional treatment particularly when detected at an earlier stage. About 10-22% of the patients develops distant metastatic disease and conventional treatment is ineffective in about half of these patients [3]. In patients with distant metastasis refractory to conventional treatment, expected survival declines rapidly [4,5].

Sorafenib is an multi-target tyrosine kinase inhibitor that is given orally. It targets VEGFR 1-3 (vascular endothelial growth factor receptors 1-3), BRAF (B-type Raf kinase) and RET (rearranged during transfection) tyrosine kinase. It has an effect on proliferation of tumor cells and angiogenesis [6]. Thyroid cancers are highly vascular with over expression of VEGFR on their cells [7]. This provide the rationale for use of sorafenib in radioiodine refractory differentiated thyroid cancers.

The cutaneous manifestations of sorafenib include rash, desquamation, hand foot skin reactions, pruritus, alopecia and erythema [8]. We report the first case of hypopigmentation related to sorafenib therapy.

## Case presentation

A 46 years male presented with on and off hemoptysis and throat irritation for two years followed by voice change for four months. Examination revealed hard nodule of two cms on left lobe of thyroid gland with no palpable lymphadenopathy and normal thyroid function test. Laryngoscopy showed a mass in subglottic region with mobile vocal cords and adequate airways. C.T scan showed 4 x 3 cm mass in left lobe of thyroid extending into left paralaryngeal space, prevertebral fascia on left side with destruction of cricoid cartilage and enlargement of delphian node. Thyroid technetium scan revealed cold nodule in left lobe which came out to be a papillary carcinoma thyroid on fine needle aspiration cytology (FNAC). He had total thyroidectomy with excision of paratracheal mass. Histopathology confirmed papillary carcinoma thyroid. Stage was PT4, Nx, Mx with no tumor identified in paratracheal tissue. Subsequently he received 100 mci of RAI^131^for remnant ablation. Post ablative whole body scan (WBS) revealed no distant metastasis. Six months later he had stimulated thyroglobulin (sTg) of 2476 ng/ml with negative anti thyroglobulin antibodies (Anti Tg) and his neck ultrasound revealed recurrent lesion of 1.9 x 1.4 cms with metastatic lymph nodes in neck confirmed by subsequent CT Scan. He received RAI^131^ 150 mci as the initial surgeon declined further surgery and post therapeutic WBS revealed functioning thyroid tissue in neck with no nodal or distant metastasis.

One year later his sTg was 2013 ng/ml with negative Anti Tg and diagnostic WBS revealed residual disease in thyroid bed with no nodal or distant metastasis. His CT scan revealed heterogeneously enhancing thyroid mass in left lobe measuring 3.6 x 3.6 cms with one centimeter sized right supraclavicular and left level V lymph nodes. He had excision of thyroid recurrence and left level VI lymph nodes. Histopathology confirmed papillary Carcinoma thyroid with lymph node metastasis. He then received further 100 mci of RAI^131^. Seven months later his STg was 2328 ng/ml, anti Tg was negative and ultrasound revealed residual/recurrent disease in left lobe of thyroid while his diagnostic whole body scan (WBS) was negative. CT scan revealed residual thyroid disease with multiple enlarged lymph nodes(left supraclavicular, left level V, left level III) with metastatic nodules in both lungs. Another dose of RAI^131^ 120 mci given. Post therapeutic WBS revealed increased uptake over thyroid bed and left cervical region but no uptake in the lung or elsewhere in the body.

**Figure 1:**
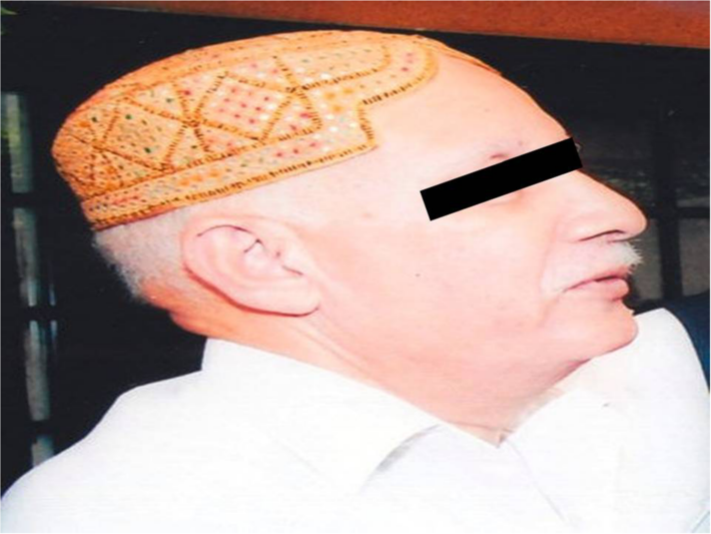
Photograph of the patient while on sorafenib treatment showing hypopigmentation of face, hair loss over scalp, eye brows and moustache.

**Figure 2:**
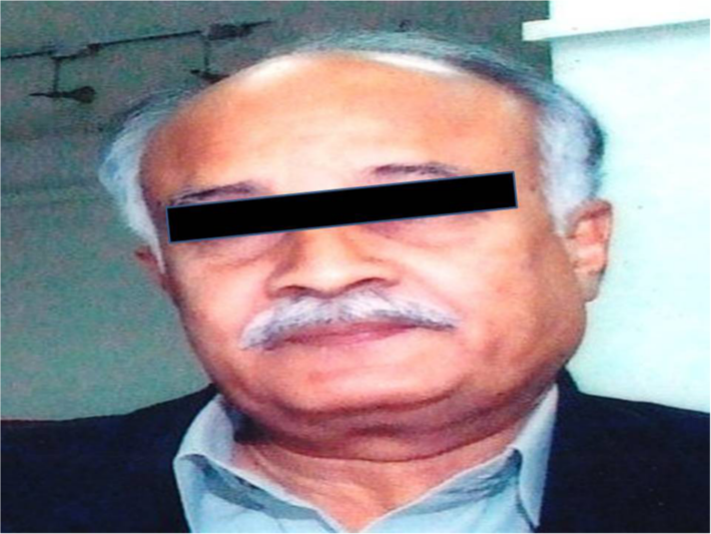
Photograph of same patient six weeks after discontinuation of sorafenib showing return of his normal skin colour with regaining of hairs over scalp, eye brows and moustache.

Despite receiving RAI^131^ 530 mCi cumulative dose, patient had persistent disease with lung metastasis, therefore a TKI, sorafenib at a recommended dose of 400 my twice daily was started. His unstimulated thyroglobulin was 373 before starting sorafenib and dropped to 80.70 in 6 months. Initially after starting sorafenib he developed diarrhea which was initially whitish,watery and later became solid. Its frequency was 6-8/day. Six weeks after starting sorafenib he developed hypopigmentation of skin of the whole body more marked on the face and also had hair loss over scalp, eye brows and moustache (Figure 1). He also experienced desquamation of hands and feet that was non pruritic in nature and mainly on sides of fingers, toes and at heels. Patient was not having any coexisting disease and there was no history of any other drug intake. He took sorafenib for 6 months. As there was no other cause thought to be the culprit and patient was very much concerned with hypopigmentation therefore Sorafenib was discontinued as patient preferred it instead of reducing the dose. His diarrhea subsided spontaneously a week later and his skin became normalized spontaneously four weeks later (Figure 2).

## Discussion

Sorafenib is a oral tyrosine kinase inhibitor. It is effective in patients with advanced Hepatocellular carcinoma and Renal cell carcinoma [9,10]. It is also effective in patients with radioiodine refractory advanced thyroid cancers as demonstrated in one phase III and three phase II clinical trials [11-14]. These clinical trials with Sorafenib in metastatic Differentiated thyroid cancers have shown efficacy in metastatic radioiodine refractory differentiated thyroid cancers [11-14]. Scheider et al conducted a clinical trial with Sorafenib in 31 radioactive iodine refractory thyroid cancer patients and found a median progression free survival (PFS) of 18 months, median overall survival (OS) of 34.5 months with a partial response (PR) of 31% [11]. Gupta et al conducted a clinical trial with Sorafenib in 30 radioactive iodine refractory thyroid cancer patients and found a median progression free survival (PFS) of 79 weeks, median overall survival (OS) of 84 weeks with a partial response (PR) of 23% [12]. Kloos et al conducted a clinical trial with Sorafenib in 41 papillary thyroid cancer patients and found a median progression free survival (PFS) of 15 months with a partial response (PR) of 15% [13]. Brose MS et al conducted a phase III DECISION trial with Sorafenib in 417 patients having radioiodine resistant advanced differentiated thyroid cancer and found a PFS 10.8 months [14].

Sorafenib is associated with high frequency of dermatological side effects which includes rash, desquamation, hand foot skin reactions, pruritus, alopecia, subungual splinter hemorrhages, alopecia, xerosis, scalp dysesthesia, stomatitis and facial erythema [8,10,15-17]. Facial erythematous eruptions is the most frequent cutaneous adverse manifestation associated with sorafenib, its overall incidence is 63% [15]. It usually appears 1 to 3 weeks after starting treatment. Treatment of this side effect is symptomatic as it resolves spontaneously. Hand-foot skin reaction (HFSR) is the 2^nd^ most frequent cutaneous adverse manifestation associated with sorafenib, its overall incidence is 30% to 60% [10,15-17]. It usually appears after 2 to 3 weeks of treatment [8]. Patients usually present with tingling or painful sensations with a symmetric red areas on palms and soles alongwith an intolerance to contact with cold objects. These symptoms may affect walking. Frequently these patients present with hyperkeratosis. Treatment is symptomatic with or without dose reduction/interruption. Scalp dysesthesia is found in 49% of the patients treated with sorafenib [15]. It usually appears early within 3 weeks of starting sorafenib and resolved spontaneously in few weeks. Alopecia with or without body hair loss found in 26 to 44% of patients treated with sorafenib [10,15,16]. It appears within 2 to 16 weeks after starting treatment [15,16]. Hairs regrow spontaneously with or without sorafenib discontinuation. Painless Subungual splinter hemorrhages are reported in 70% sorafenib treated patients [15]. They present as straight black or red lines under the nails after 1 to 2 weeks of treatment and resolve spontaneously [8,15]. Stomatitis is reported in 19 to 26% of sorafenib treated patients [15,16]. It appears within fist eight weeks of treatment and require either dose reduction or transient drug interruption. Other cutaneous manifestations of sorafenib includes Xerosis in 8 to 23% and Pruritus in 8 to 19% of sorafenib treated patients [10,15,17] and the treatment is usually symptomatic.

To our knowledge, hypopigmentation in our patient appears to be the first reported of its kind in the literature to date. It appeared six weeks after starting sorafenib. It was generalized but more marked over face. Our patient took sorafenib for alltogether 6 months. His skin became normalized in four weeks after discontinuation of sorafenib. The most important complication of hypopigmentation is photosensitivity that can be treated with avoidance of sun exposure and use of sun blocks.

## Conclusion

Sorafenib is used in Renal cell carcinoma, Hepatcellular carcinoma and radioactive iodine refractory thyroid carcinoma therefore it is very important to be aware of hypopigmentation as a potential side effect for both physicians and patients. Further studies are needed to unveil the pathophysiology of this unique side-effect of hypopigmentation with sorafenib in differentiated thyroid cancer patients.

### Consent

“Written informed consent was obtained from the patient for publication of this Case report and any accompanying images. A copy of the written consent is available for review by the Editor-in-Chief of this journal”.

## Abbreviations

TKI: Tyrosine kinase inhibitor; DTC: Differentiated thyroid carcinoma; RAI131: Radioactive iodine; VEGFR: Vascular endothelial growth factor receptors; BRAF: B-type Raf kinase and; RET: Rearranged during transfection; WBS: Whole body scan; sTg: Stimulated thyroglobulin; Anti Tg: Anti thyroglobulin antibodies.

## Competing interests

The authors declare that they have no competing interests.

## Authors’ contributions

SZ led the conception and design, acquisition of data, review of literature, and drafted the manuscript. AA reviewed the manuscript. MI reviewed the manuscript. NI gave the concept of research paper, and critically reviewed the manuscript. All authors read and approved the manuscript.

## Authors’ information

• SZ is member of the Royal Colleges of Physicians of the United Kingdom. He is Fellow in Endocrinology, Diabetes & Metabolism, Department of Medicine, Aga Khan University Hospital. He was involved in the medical management of the patient.

• AA is member of the Royal Colleges of Physicians of the United Kingdom. He is Fellow in Endocrinology, Diabetes & Metabolism, Department of Medicine, Aga Khan University Hospital. He was also involved in the medical management of the patient.

• MI is fellow of the College of Physicians & Surgeons of Pakistan. He is Associate Professor in Otorhinolaryngology, Department of Surgery, Aga Khan University Hospital. He was patient’s primary surgeon.

• NI is fellow of the Royal College of Physicians of London. He is Professor in Diabetes, Endocrinology & Metabolism, Department of Medicine, Aga Khan University Hospital. He is also the Director, Diabetes, Endocrinology and Metabolism Fellowship Program, Aga Khan University Hospital. He was patient’s primary physician & endocrinologist.

## Pre-publication history

The pre-publication history for this paper can be accessed here:

http://www.biomedcentral.com/1472-6823/13/29/prepub
